# Molecular mechanism of Ganji Fang in the treatment of hepatocellular carcinoma based on network pharmacology, molecular docking and experimental verification technology

**DOI:** 10.3389/fphar.2023.1016967

**Published:** 2023-01-19

**Authors:** Miaolun Yang, Qian Yan, Yuehua Luo, Boqing Wang, Shicong Deng, Huiyan Luo, Baoqian Ye, Xiongwen Wang

**Affiliations:** ^1^ The First Clinical Medical School, Guangzhou University of Chinese Medicine, Guangzhou, China; ^2^ The First Affiliated Hospital, Guangzhou University of Chinese Medicine, Guangzhou, China

**Keywords:** Ganji Fang, hepatocellular carcinoma, network pharmacology, HepG2 cells, PI3K/Akt signaling pathway, EPHA2

## Abstract

**Background:** Hepatocellular carcinoma (HCC) is a malignant tumor harmful to human health. Ganji Fang (GJF) has good clinical efficacy in the treatment of HCC, but its mechanism is still unclear.

**Objective:** The aim of this study was to investigate the mechanism of action of GJF in the treatment of HCC through network pharmacology, molecular docking and *in vitro* experiments.

**Methods:** A series of network pharmacology methods were used to identify the potential targets and key pathways of GJF in the treatment of HCC. Then, molecular docking technology was used to explore the binding ability of key active ingredients and targets in GJF. Multiple external databases were used to validate the key targets. In in vitro experiments, we performed MTT assays, wound-healing assays, cell cycle assays, apoptosis assays and RT‒qPCR to verify the inhibitory effect of GJF on the Human hepatoma G2 (HepG2) cells.

**Result:** A total of 162 bioactive components and 826 protein targets of GJF were screened, and 611 potential targets of HCC were identified. Finally, 63 possible targets of GJF acting on HCC were obtained. KEGG enrichment analyses showed that the top five pathways were the cell cycle, cellular senescence, p53 signaling pathway, PI3K/Akt signaling pathway, and progesterone-mediated oocyte maturation. Among them, we verified the PI3K/Akt signaling pathway. CCNE1, PKN1, CCND2, CDK4, EPHA2, FGFR3, CDK6, CDK2 and HSP90AAI were enriched in the PI3K/Akt pathway. The molecular docking results showed that the docking scores of eight active components of GJF with the two targets were all less than -5.0, indicating that they had certain binding activity. *In vitro* cell experiments showed that GJF could inhibit the proliferation and migration of HepG2 cells, block the cell cycle and induce apoptosis of HepG2 cells, which may be related to the PI3K/Akt signaling pathway. In summary, EPHA2 may be an important target of GJF in HCC, and pachymic acid may be an important critical active compound of GJF that exerts anticancer activity.

**Conclusion:** In general, we demonstrated, for the first time, that the molecular mechanism of GJF in HCC may involve induction of G0/G1 phase cycle arrest through inhibition of the PI3K/Akt signaling pathway and promote apoptosis of hepatoma cell lines. This study provides a scientific basis for the subsequent clinical application of GJF and the in-depth study of its mechanism.

## Graphical Abstract

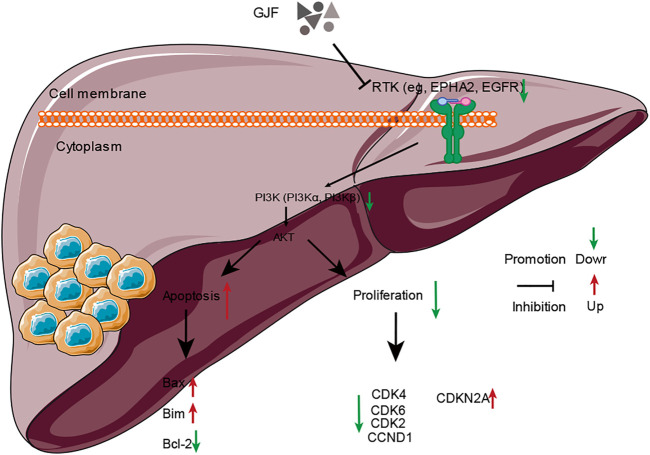

## 1 Introduction

Primary liver cancer (PLC) includes hepatocellular carcinoma (HCC) and intrahepatic cholangiocarcinoma as well as other rare types (Bray et al., 2018). HCC, the most prevalent type of PLC, accounts for approximately 90% of cases (Gunasekaran et al., 2020). HCC ranks fifth in terms of global cases and second in terms of deaths for males (Bray et al., 2018). Studies have shown that the incidence of HCC has stabilized in men after decades of steep increase but continues to rise in women by >2% annually (Siegel et al., 2021). Surgical removal involving resection and transplantation remains the only curative treatment for most types of liver tumors (Petrowsky et al., 2020). In addition, chemotherapy, radiotherapies, radiofrequency ablation (RFA), percutaneous ethanol injection (PEI), transarterial chemoembolization (TACE), targeted therapy, and immunological therapy are also important treatment methods (Petrowsky et al., 2020). However, all of these treatments have side effects that affect patients’ lives. As the use of immune checkpoint inhibitors continues to expand, this treatment will become more crucial than early detection, and management of these side effects becomes paramount to maximize the duration of treatment while minimizing toxicity for patients (Hussaini et al., 2021).

Although the effect of traditional Chinese medicine (TCM) on tumor shrinkage is not as obvious as that of modern Western medicine, reducing toxicity and increasing the efficacy of TCM can help to reduce the side effects, improve the curative effect and improve the quality of life of patients (Z. Ma et al., 2019; Ye et al., 2015). Evidence-based functions of Chinese herbal medicines against cancer can be summarized as enhancement of natural killer (NK) cell activity or relative percentage; prevention of tumor growth and metastasis; relief of side effects or complications of therapeutic strategies (i.e., chemotherapy, radiotherapy, and resection) (H. Liu et al., 2021). Some studies have shown that adjuvant therapy with TCM may prolong the median overall survival time and reduce the mortality of HCC patients (X. Liu et al., 2019). GJF is an established prescription drug with beneficial gas spleen detoxification and anticancer functions. GJF is commonly used to treat HCC in clinical practice, and some patients with advanced liver cancer even choose to be treated with pure Chinese medicine. Previous clinical and animal studies have preliminarily explored whether GJF may have anti-inflammatory effects, protect the liver, inhibit tumor cell proliferation, and induce tumor cell apoptosis. Therefore, this study will further explore the clinical application value and possible mechanism of GJF along the lines of previous research. TCM compounds are difficult to study because they are composed of multiple herbs, contain complex components, and their mechanism of action is unclear.

**FIGURE 1:**
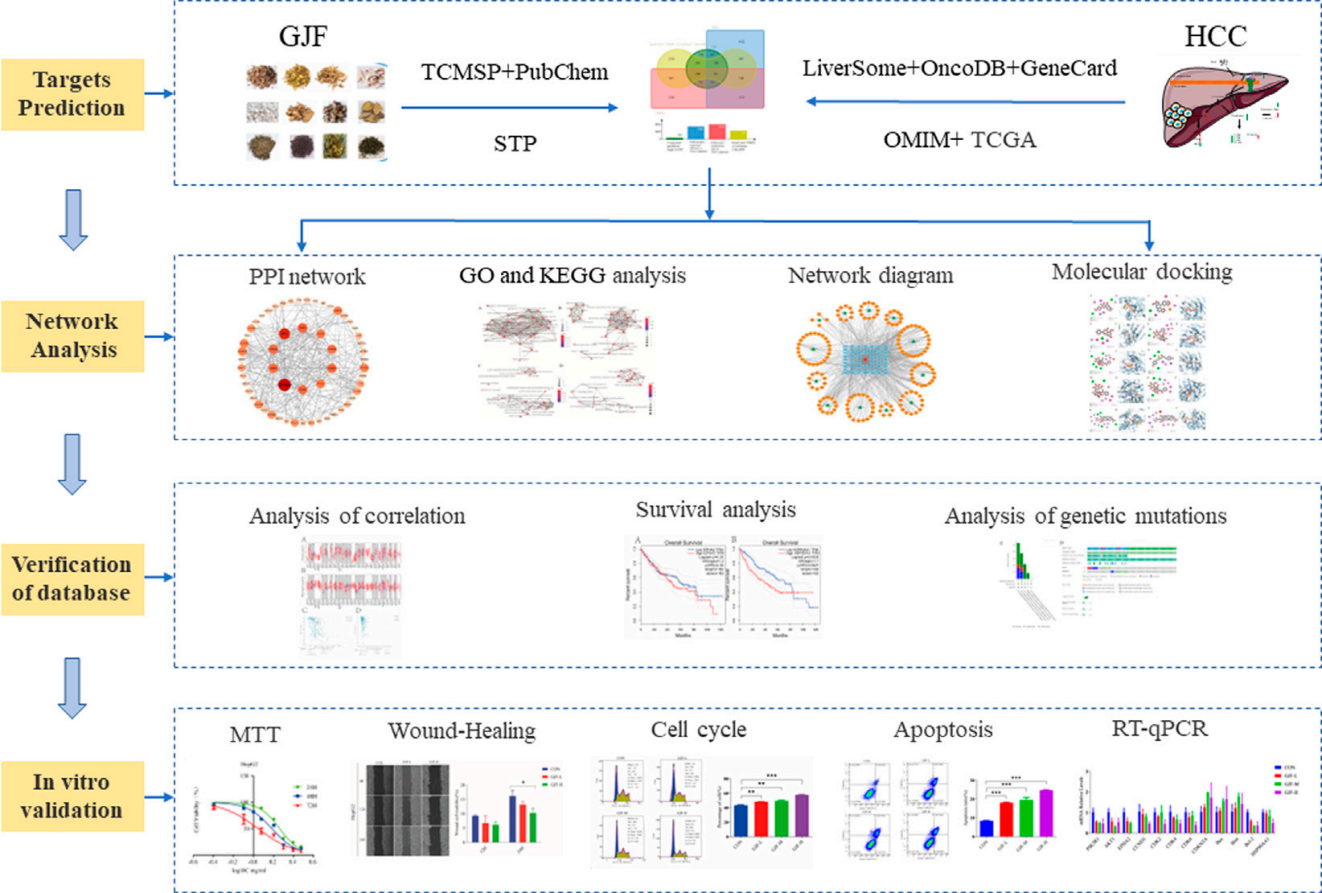
Overall workflow of this study (GJF, Ganji Fang; TCMSP, Traditional Chinese Medicine System Pharmacology database; STP, SwissTargetPrediction database; HCC, Hepatocellular carcinoma).

Network pharmacology uses drug, compound, gene, and disease database information to construct drug-target, target-disease, and drug-disease interaction networks to reveal the complex mechanisms of TCM formulations that have multiple targets and multicomponent characteristics (S. Li & Zhang, 2013), which provides a new strategy for the study of complex traditional Chinese medicine systems. The comprehensive, systematic and holistic nature of network pharmacology are consistent with the multicompound, multitarget and multipathway characteristics of Chinese medicine (Luo et al., 2020). Many researchers use network pharmacology to predict the active phytochemicals and molecular mechanisms of traditional Chinese medicine in the treatment of HCC. Many researchers use network pharmacology to predict the active phytochemicals and molecular mechanisms of traditional Chinese medicine in the treatment of HCC (Khan & Lee, 2022). In this paper, the possible pathways and targets of GIF for HCC treatment were predicted mainly through network pharmacology methods and then verified by molecular docking, external databases, experimental research and other methods. The detailed flow chart of the study is shown in Figure 1.

## 2 Materials and methods

### 2.1 Screening active compounds GJF

We obtained the compounds of each drug of GJF from the Traditional Chinese Medicine System Pharmacology Database and Analysis Platform (TCMSP, http://lsp.nwu.edu.cn/tcmsp.php, accessed on 12 May 2020). In addition, the potential active compounds of each Chinese medicine were also supplemented through a literature search. Oral bioavailability (OB) and drug-likeness (DL) are some of the most important pharmacokinetic parameters in the process of absorption, distribution, metabolism, and excretion (ADME). We screened compounds on the condition that oral utilization of compounds OB ≥ 30% and DL ≥ 0.18.

### 2.2 Prediction of drug targets for GJF

The 3D structures of the active ingredients of each compound and canonical SMILES format were downloaded from the PubChem database (https://pubchem.ncbi.nlm.nih.gov/, accessed on 20 May 2020). The potentially effective action targets of GJF were obtained using the SwissTargetPrediction database (STP, http://www.swisstargetprediction, accessed on 20 May 2020) (Daina et al., 2019). We chose the target probability to be greater than 0, and if there were too many predicted targets, we chose the top 100 targets ranked according to the target probability. When SwissTargetPrediction could not predict relevant targets, we used the TMSCP database to make a supplement prediction.

### 2.3 Extracting the targets of hepatocellular carcinoma

We consolidated HCC-related targets through the Databases Liverome (http://liverome.kobic.re.kr/index.php, accessed on 23 May 2020), OncoDB. HCC databases (http://www.oncodb.hcc.ibms.sinica.edu.org/, accessed on 23 May 2020), GeneCards databases (https://www.genecards.org/, accessed on 23 May 2020), and OMIM databases (http://www.ncbi.nlm.nih.gov/, accessed on 23 May 2020) (Su et al., 2007; Lee et al., 2011; Paolacci et al., 2019).

Additionally, the mRNA sequencing data of HCC were downloaded for the PI3K/Akt signaling pathway on The Cancer Genome Atlas (TCGA) dataset (http://cancergenome.nih.gov/, accessed on 23 May 2020), including 370 primary HCC tissues and 50 normal tissues and the corresponding clinical follow-up data. To improve the accuracy of the data, we preprocessed the dataset, including deleting the sites where 70% methylation levels were unavailable and samples expressing more than 30% missing values. Genes with zero RPKM expression in the sample were also excluded (J. Xu et al., 2015).

The data were standardized using the RMA algorithm in the “Limma” package in R software (Smyth, 2004; Law et al., 2014; Ritchie et al., 2015). A false detection rate (FDR) method was used to regulate the *p-value*. The screening criteria for differentially methylated genes (DMGs) were FDR <0.05 and β values >0.2, HCC in cancerous tissue and normal liver tissue differentially expressed genes (Differentially expressed genes, DEGs) in line with∣1og _2_ (fold change)∣> 1 and FDR <0.05 standard. We intersected HCC-related targets in the public database with DMGS and DEGs in the TCGA database, regarded this intersection as possible key targets in HCC, and mapped these targets to related targets predicted by compounds in GJF as potential targets of GJF for HCC.

### 2.4 Construction of networks and analysis

The key targets of GJF for HCC were imported into the STRING database to construct a PPI network, and the confidence level was set as ≥0.70. Cytoscape (Version: 3.9.1) software was used for visualization. The key genes and their relationship network of TCM, drug active ingredient, and drug and disease crossing were imported into Cytoscape 3.9.1 software to construct a herb-compound-target network of GJF.

### 2.5 Gene ontology and pathway enrichment analysis for HCC-Related targets of GJF

Gene Ontology (GO) and Kyoto Encyclopedia of Genes and Genomes (KEGG) enrichment analyses were performed using the “cluster profile” package in R software for better biological interpretation (Yu et al., 2012). GO and KEGG enrichment analyses of the potential targets were performed using the database for annotation, visualization, and integrated discovery (DAVID, https://david.ncifcrf.gov/, accessed on 26 May 2021). GO enrichment analysis included biological processes (BP), molecular functions (MF), and cellular components (CC).

### 2.6 Molecular docking

The RCSB PDB database (https://www.rcsb. org/, accessed on 10 June 2021) to retrieve and download 3D structure files for key target proteins, while using the PubChem database (https://pubchem.ncbi.nlm.nih.gov/, accessed on 12 June 2021) to download 3D structure files for active compounds. For docking analysis, all protein and molecular files were converted into PDBQT format with all water molecules excluded, and polar hydrogen atoms were added. The grid box was centered to cover the domain of each protein and to accommodate free molecular movement. The grid box was set to 30 Å × 30 Å × 30 Å, and the grid point distance was 0.05 nm. Molecular docking studies were performed by Autodock Vina 1.2.2 (http://autodock.scripps.edu/, accessed on 12 June 2021).

### 2.7 Validation of external databases

For the above key targets, we performed a series of validations in the TCGA database. The cBioPortal (http://cbioportal.org, accessed on 16 June 2021) genomics database (J. Gao et al., 2013) demonstrated the association of genetic mutation status, mRNA, and DNA methylation. The GEPIA (Gene Expression Profiling Interactive Analysis, http://gepia.cancer-pku.cn/, accessed on 16 June 2021) database was used to examine the prognostic differences between high and low gene levels. The (T. Li et al., 2017) database was used to verify the difference in mRNA level expression.

### 2.8 *In Vitro* validation cell culture

The HCC cell line HepG2, which was purchased from Guangzhou Cellcook Biotech Co., Ltd., was routinely cultured in DMEM (Gibco, Grand Island, NY, United States) containing 10% FBS (Thermo Fisher Scientific, Rochester, NY, United States). Cells were cultured at 37 °C in a 5% CO^2^ incubator, subcultured at a ratio of 1:2, and used for subsequent experiments after reaching the logarithmic growth phase. In this study, we set the 72-hour IC_50_ to a medium dose, half the 72-hour IC_50_ to a low dose, and two times the 72-hour IC_50_ to a high dose.

### 2.9 The source of GJF and preparation of lyophilized powder

GJF was purchased from the Outpatient Pharmacy of the First Affiliated Hospital of Guangzhou University of Traditional Chinese Medicine. The dose of 1 fu of GJF was 241 g (12 g bupleurum, 12 g paeoniae, 30 g codonopsis, 15 g poria, 15 g atractylodes, 20 g Rhizoma lucidum, 30 g ligustrum scutellariae, 12 g Scutellaria scutellariae, 20 g Rhizoma barbiliae, 30 g Augustaloza, 15 g Zedoary Zedoaria, 30 g Pellucidaria). Deionized water was added at 10x (approximately 2400 ml), sampled was cooked twice and centrifuged at 1000 r for 5 min. The supernatant was collected and refrigerated at -80 °C for 6–8 h until it was completely frozen. Then, the samples were quickly put into a lyophilized powder machine. After the preparation of lyophilized powder, complete culture medium was used to prepare 50 mg/ml liver-product solution.

### 2.10 MTT assay

The logarithmic phase cells were collected, the cell density was adjusted to 5 ˣ 10^4^/ml, and the cells were inoculated in three 96-well plates. Then, 100 µL of GJF solution with different concentration gradients was added to the cell culture box. One plate was removed at 24 h, 48 h, and 72 h, and a mixture of MTT solution and basic medium (ratio: 1:4) was added under closed light conditions, 100 µL for each well. After further incubation for 4 h, 150 µL DMSO solution was added to each well. The wavelength of the enzyme plate analyzer was set at 490 nm, and the OD values of 3 96-well plates were measured at different times.

### 2.11 Wound-healing assay

HepG2 cells in logarithmic growth were cultured in a 6-well plate (2 × 10^5^ cells/well). After the cells reached a subconfluency state (approximately 80%), a 2-mm scratch was made along the midline of the plate. Cells were assigned to the blank serum group (blank serum) (cells cultured with normal culture medium supplemented with blank serum), GJF low-dose group (GJF-L), and GJF high-dose group (GJF-H), with two replicate wells set up for each group. When cells were cultured for 0, 12, and 24 h in drug-containing or drug-free media, they were photographed under an inverted microscope (Olympus, Tokyo, Japan), and the rate of cell migration was calculated.

### 2.12 Cell cycle detection

The cells were inoculated in a 6-well plate, divided into control group, GJF of low, medium, and high concentration group, allowed to adhere and grown to a certain density, and then a different concentration of GJF frozen-dried powder solution was added to each well. Twenty-four hours later the cells were added to 1 ml of ice bath precooled with 70% ethanol and fixed at 4 °C for 2 h or more. Then, the cells were precipitated and resuspended. Next, 0.5 ml of cyclosome iodide dyeing liquid was added to each group, incubated at 37 °C in a bath for 30 min in the dark, and stored at 4 °C or on an ice bath for 24 h to complete the flow test.

### 2.13 Apoptosis detection

The cells at several stages were inoculated in a 6-well plate, divided into control group, GJF of low, medium, and high concentration group, 24 h after taking 5–10 million suspended cells, the cells were centrifuged at 1000r for 5 min, after discarding, 195 µL Annexin V- FITC binding fluid was added to gently resuspend the cells, 5 µL Annexin V-FITC was added, 10u1 iodide dye was added, and cells were incubated at room temperature (20°C–25°C) for 10–20 min, then placed in an ice bath. The cells were tested on a flow cytometer as soon as possible within an hour.

### 2.14 RT‒qPCR microarray analysis

Total RNA was extracted from experimental cells using a universal RNA extraction kit. Then, EVO-M-MLVRT Master Mix was used for reverse transcription into cDNA. The reaction system was prepared according to the instructions of the SYBR premixed RT‒qPCR kit for RT‒qPCR amplification. The RT‒qPCR conditions were as follows: pre-denaturation at 95 °C for 30 s, denaturation at 95°C for 30 s, and annealing at 60 °C for 30 s, for a total of 40 cycles. The experiment was repeated 3 times. Gene expression was evaluated by RT-qPCR, using the 2 -∆∆CT method.

### 2.15 Statistical analysis

Measurement data are expressed as the mean taxi standard deviation (X ± S) and were statistically processed using SPSS (version: 26.0) software. When the experimental data met the normal distribution and the variance was consistent, one-way analysis of variance and two-way analysis of variance were used, and a non-parametric test was used if the data did not meet the normal distribution. *p* < 0.05 indicates that the difference is statistically significant. The above experiments were repeated three times, with the results of the cell cycle and apoptosis experiments presented in FlowJo (version 10.7.1) and the rest in GraphPad Prism (version 8.0).

## 3 Results

### 3.1 Active ingredients and prediction of a protein target of GJF

A total of 162 bioactive components and 826 protein targets of GJF were obtained from both TCMSP databases and a literature search (Supplementary Table S1). Radix Bupleuri was supplemented with Saponin A (Wen-Sheng, 2003) and Saponin D (Ren et al., 2019), Atractylodide I, Atractylodide II, and Atractylodide III were added to Atractylodes macrocephala Koidz (Zhu et al., 2018), raffinose was added to Codonopsitis Radix (S. Gao et al., 2019), Paeoniae Radix Alba was supplemented with oxypaeoniflorin, (M. Lu et al., 2019), Eclalbasaponin I and ursolic acid were added to Eclipse Herba (Q. M. Liu et al., 2012), oleanic acid was added to Fructus Ligustri Lucidi, Phytodolor and rosmarinic acid were added to Herba Sarcandrae, Scutellariae Barbatae Herba was supplemented with scutellarin, curcumin was added to Curcumae Rhizoma, and Akebiae Frucyus was added to Calceolarioside B.

### 3.2 Prediction results of hepatocellular carcinoma targets

**FIGURE 2:**
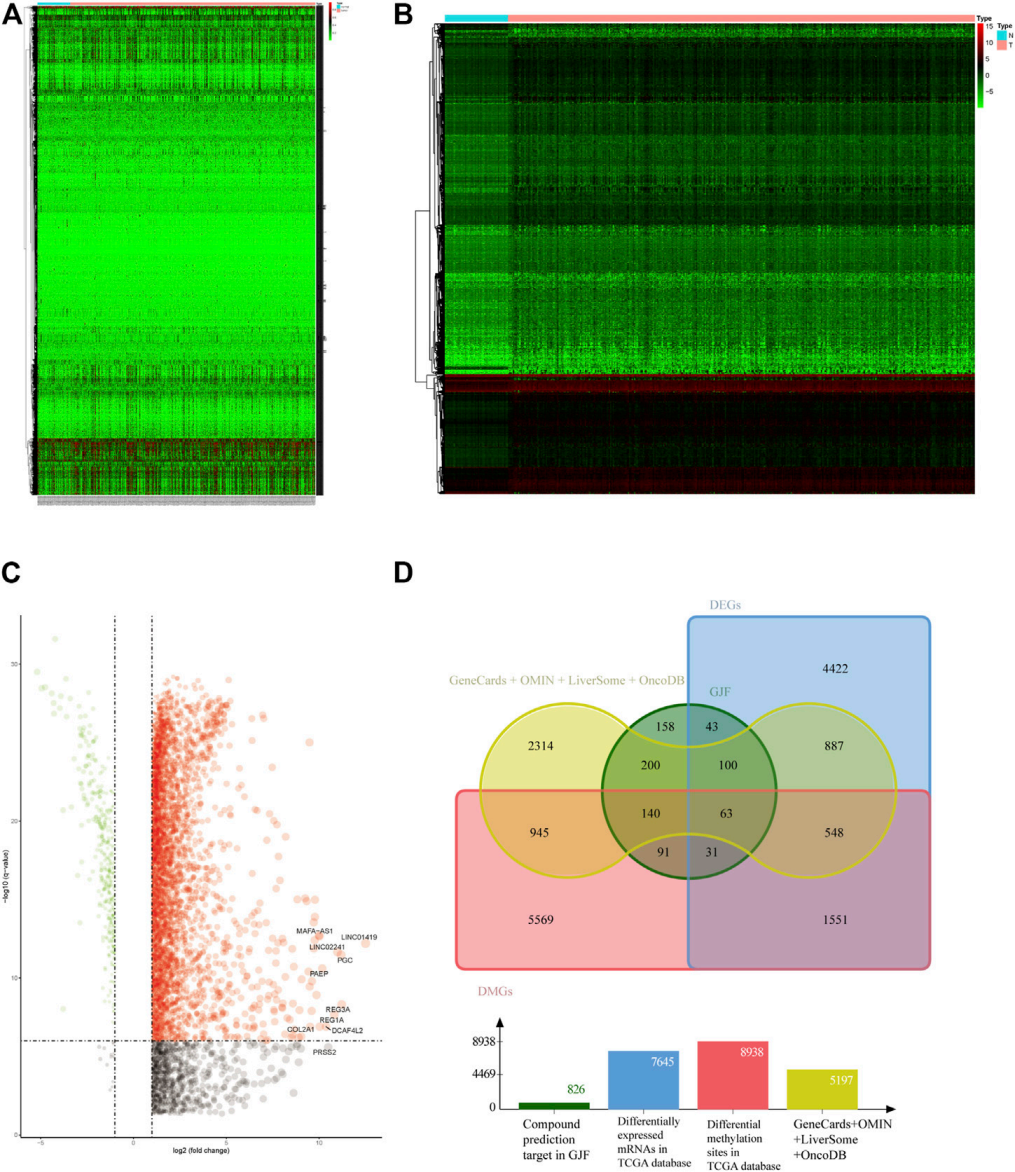
**(A)** Heatmap of HCC-associated differential methylation sites in the TCGA database **(B)** Heatmap of HCC-related differentially expressed genes in the TCGA database **(C)** Volcano plot of HCC-related differentially expressed genes in the TCGA **(D)** Venn diagram of the intersection of HCC-related targets (DEGs, differentially expressed genes; DMGs, differentially methylated genes).

We screened 5,714 genes in four public databases. The mRNA and methylation sites differentially expressed in the TCGA were further screened. According to the screening criteria for the differentially expressed genes, we obtained 7645 differentially expressed genes, and after the conversion of the differentially methylated sites, we identified 8938 differentially methylated genes, which we display in the form of heatmaps and volcano plots. (Figures 2A–C).

### 3.3 GO and KEGG enrichment analyses

**FIGURE 3:**
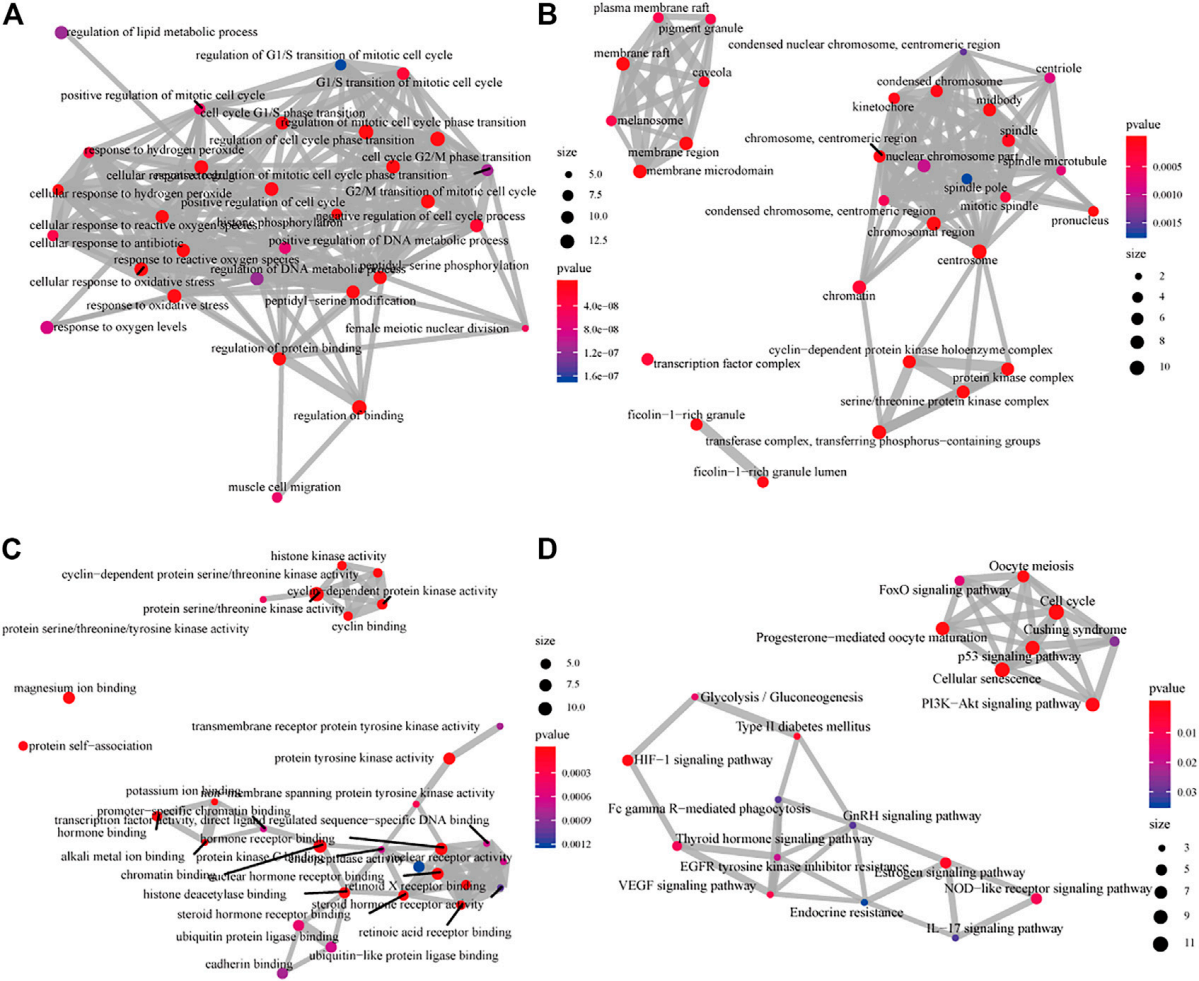
GO and KEGG analysis diagram of the intersection of targets of GJF in HCC **(A)** The analysis diagrams of the biological process **(B)** Diagrams of the analysis of cell components **(C)** The analysis diagrams of molecular functions **(D)** KEGG analysis diagram (GO, gene ontology; KEGG, Kyoto Encyclopedia of Genes and Genomes).

The 611 potential targets associated with HCC were mapped to the targets predicted by GJF (Figure 2D), and 63 intersections were obtained, which were the possible targets of GJF acting on HCC. The 63 genes were analyzed by GO and KEGG (Figure 3). According to the number of genes enriched in each item, the results showed that the top five biological processes involved in these genes were epithelial cell proliferation, regulation of epithelial cell proliferation, regulation of cell cycle phase transition, gland development, and embryonic organ development; in terms of cell composition, they were mainly involved in cell-substrate junction, cell-substrate adherens junction, focal adhesion, adherens junction, and extracellular matrix composition. In the molecular function category, they were mainly associated with cell adhesion molecule binding, proximal promoter sequence-specific DNA binding, chromatin binding, RNA polymerase II proximal promoter sequence-specific DNA binding and laminin binding. In addition, the top five pathways were the cell cycle (Gotoh et al., 2003), cellular senescence, p53, signaling pathway, PI3K/Akt signaling pathway, and progesterone-mediated oocyte maturation (Supplementary Table S2).

### 3.4 PPI network and herb-compound-target network of GJF

**FIGURE 4:**
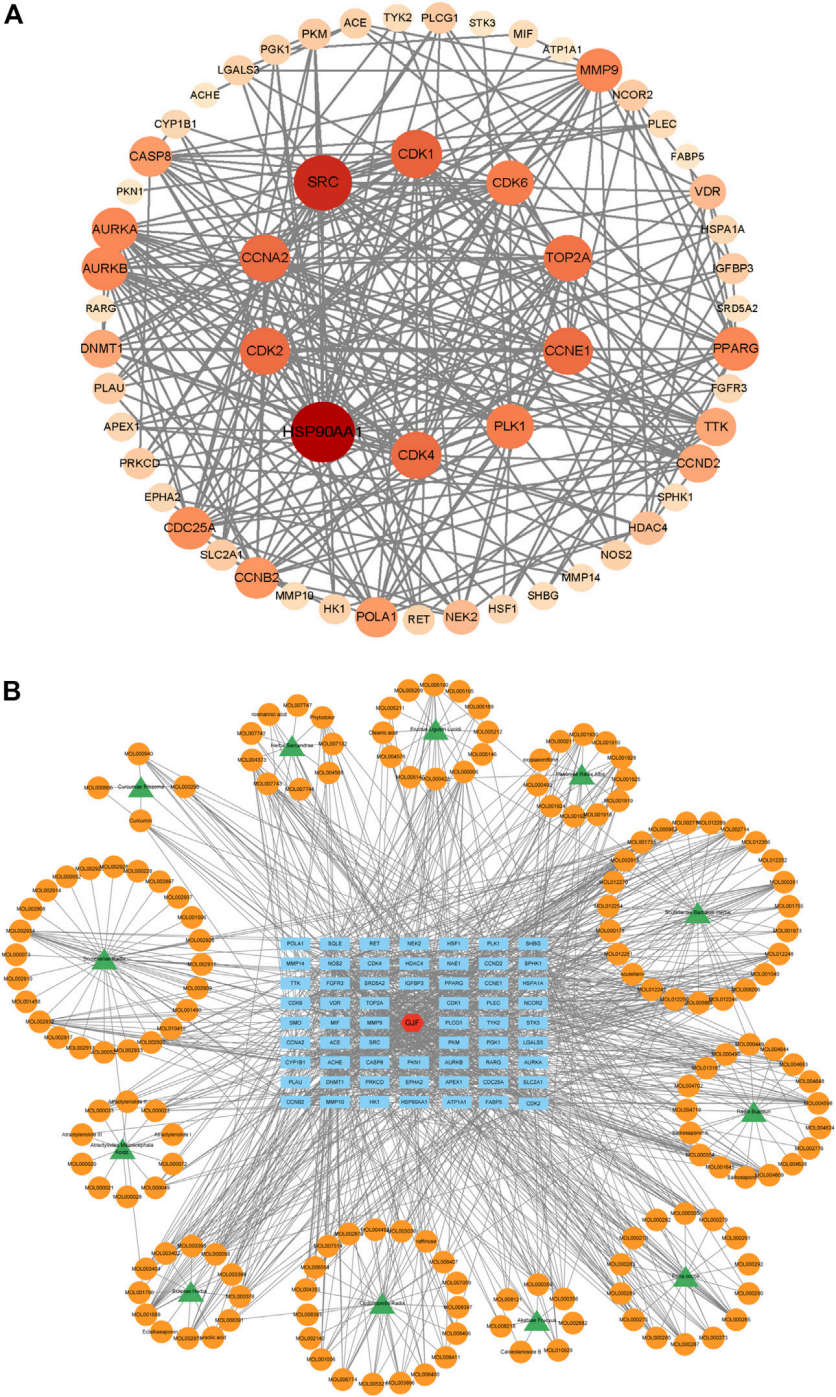
**(A)** The PPI network of 63 nodes (genes) (The darker the color, the larger the circle, the more connected; The 10 targets in the innermost layer are the key targets with the highest connection degree) **(B)** Herb-compound-target network of GJF (green represents the name of traditional Chinese medicine, orange represents the active ingredient, blue represents the key gene target, and red represents Ganji Fang).

We performed PPI network analysis of 63 intersection genes between HCC (Figure 4A). GJF and the top 10 genes were HSP90AA1, SRC, CDK1, CCNA2, CDK4, PLK1, CDK2, TOP2A, AURKB and CDK6. Based on the results of the KEGG analysis, we verified the role of the PI3K/Akt signaling pathway in GJF. KEGG analysis showed that CCNE1, PKN1, CCND2, CDK4, EPHA2, FGFR3, CDK6, CDK2, and HSP90AA1 were enriched in the PI3K/Akt pathway. We constructed a network diagram of “TCM-compound-target-pathway” (Figure 4B). The network consisted of 235 nodes (12 TCM name nodes, 162 active ingredient nodes, 63 gene target nodes) and 1225 edges.

### 3.5 Molecular docking

**FIGURE 5:**
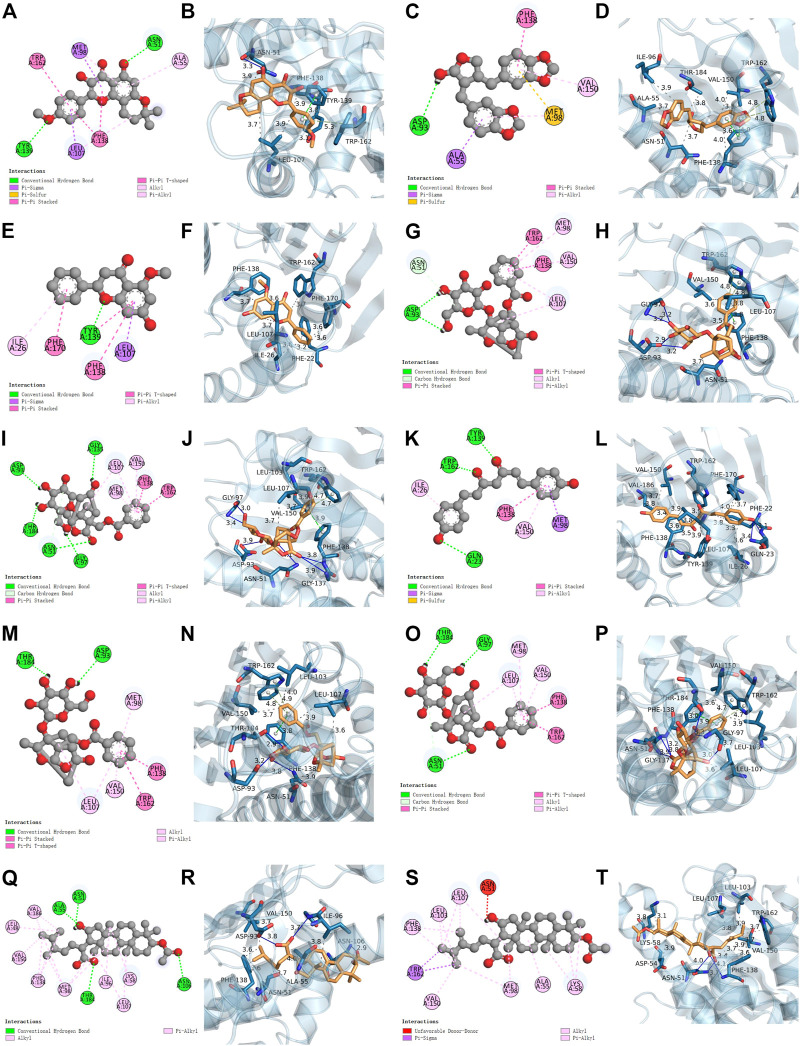
Molecular docking results. HSP90AA1 binds with MOL004373 (2D and 3D) **(A**, **B)**, MOL013187 (2D and 3D) **(C**, **D)**, MOL000228 (2D and 3D) **(E**, **F)**, MOL001924 (2D and 3D) **(G**, **H)**, MOL001928 (2D and 3D) **(I**, **J)**, MOL000940 (2D and 3D) **(K**, **L)**. EPHA2 binds with MOL001924 (2D and 3D) **(M**, **N)**, MOL001928 (2D and 3D) **(O**, **P)**, MOL000289 (2D and 3D) **(Q**, **R)**, MOL000276 (2D and 3D) **(S**, **T)**.

We conducted a molecular docking analysis between HSP90AA1, anhydroicaritin, cubebin, (2R)-7-hydroxy-5-methoxy-2-phenylchroman-4-one, paeoniflorin (PF), albiflorin_qt, and bisdemethoxycurcumin to screen the compounds with the strongest binding ability to HSP90AA1. In addition, PF, albiflorin_qt, pachymic acid (PA), and (7,9 (11)-dehydropachymic acid were used to screen the compounds that acted most closely with EPHA2. The results showed that the docking scores of the eight active components of GJF with the two targets were all less than -4.0, indicating that they had certain binding activity. As shown in Table 1, the strongest binding energy between PF and HSP90AA1 is -7.3 and that between PA and EPHA2 was -8.8. Therefore, these key components may be potential active compounds of GJF in the treatment of HCC (Figure 5).

**TABLE 1:** Docking scores of key compounds and targets in hepatocellular formulas.

Molecule name	Molecule ID	Receptor	PDB ID	Binding affinity (kcal/mol)
Anhydroicaritin	MOL004373	HSP90AA1	6lr9	−6.2
Cubebin	MOL013187	HSP90AA1	6lr9	−7
(2R)-7-hydroxy-5-methoxy-2-phenylchroman-4-one	MOL000228	HSP90AA1	6lr9	−6
Paeoniflorin	MOL001924	HSP90AA1	6lr9	−7.3
Albiflorin_qt	MOL001928	HSP90AA1	6lr9	−6.4
Bisdemethoxycurcumin	MOL000940	HSP90AA1	6lr9	−5.5
Paeoniflorin	MOL001924	EPHA2	6q7e	−8.4
Albiflorin_qt	MOL001928	EPHA2	6q7e	−8.4
Pachymic acid	MOL000289	EPHA2	6q7e	−8.8
7,9 (11)-dehydropachymic acid	MOL000276	EPHA2	6q7e	−8.5

### 3.6 Validation of EPHA2 and HAP90AA1 in the TCGA database

**FIGURE 6:**
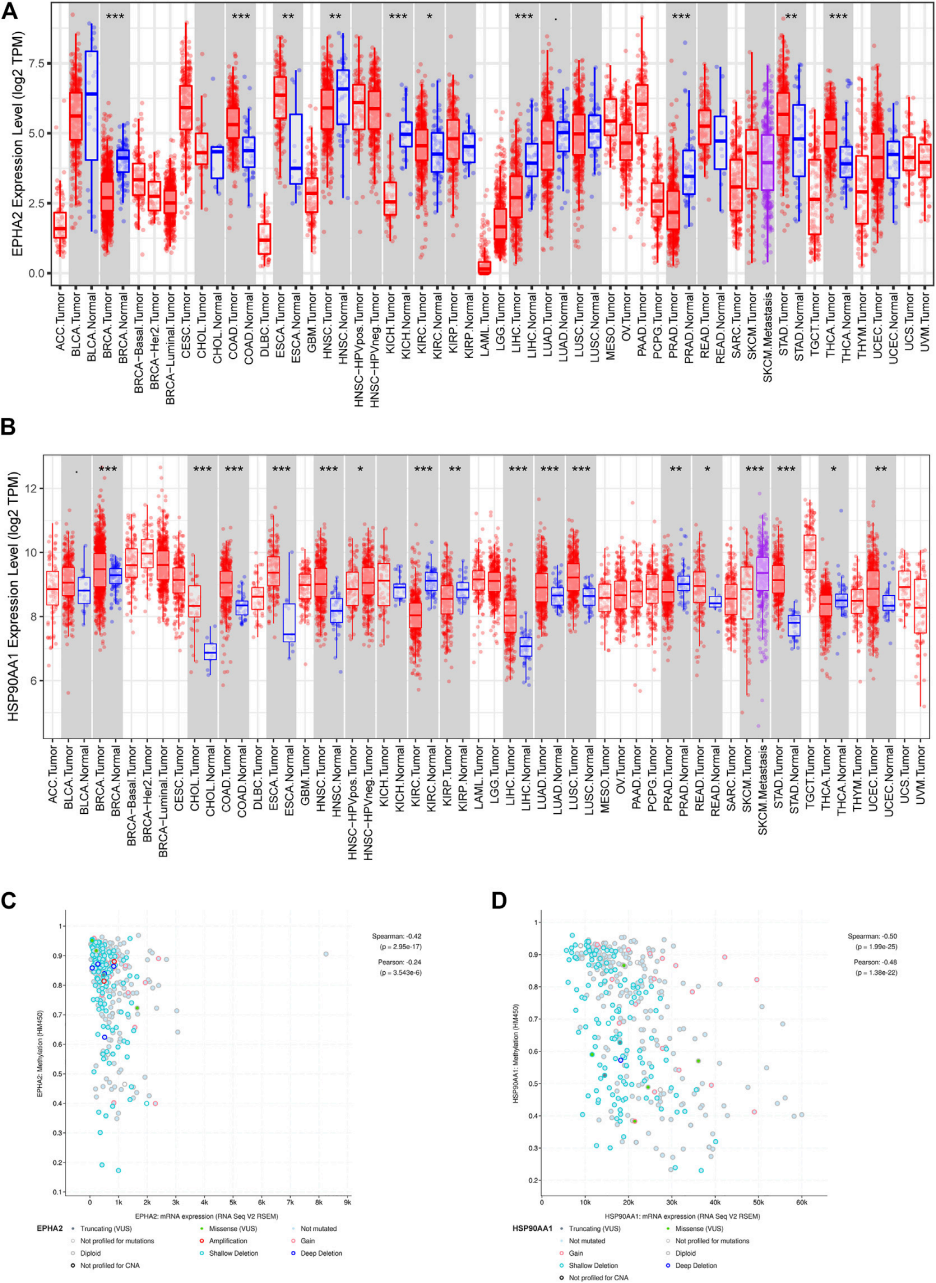
**(A, B)** Differential expression of EPHA2 and HSP90AA1 in various tumors. **(C, D)** Correlation analysis of mRNA expression levels and DNA methylation of EPHA2 and HSP90AA1 in HCC.

**FIGURE 7:**
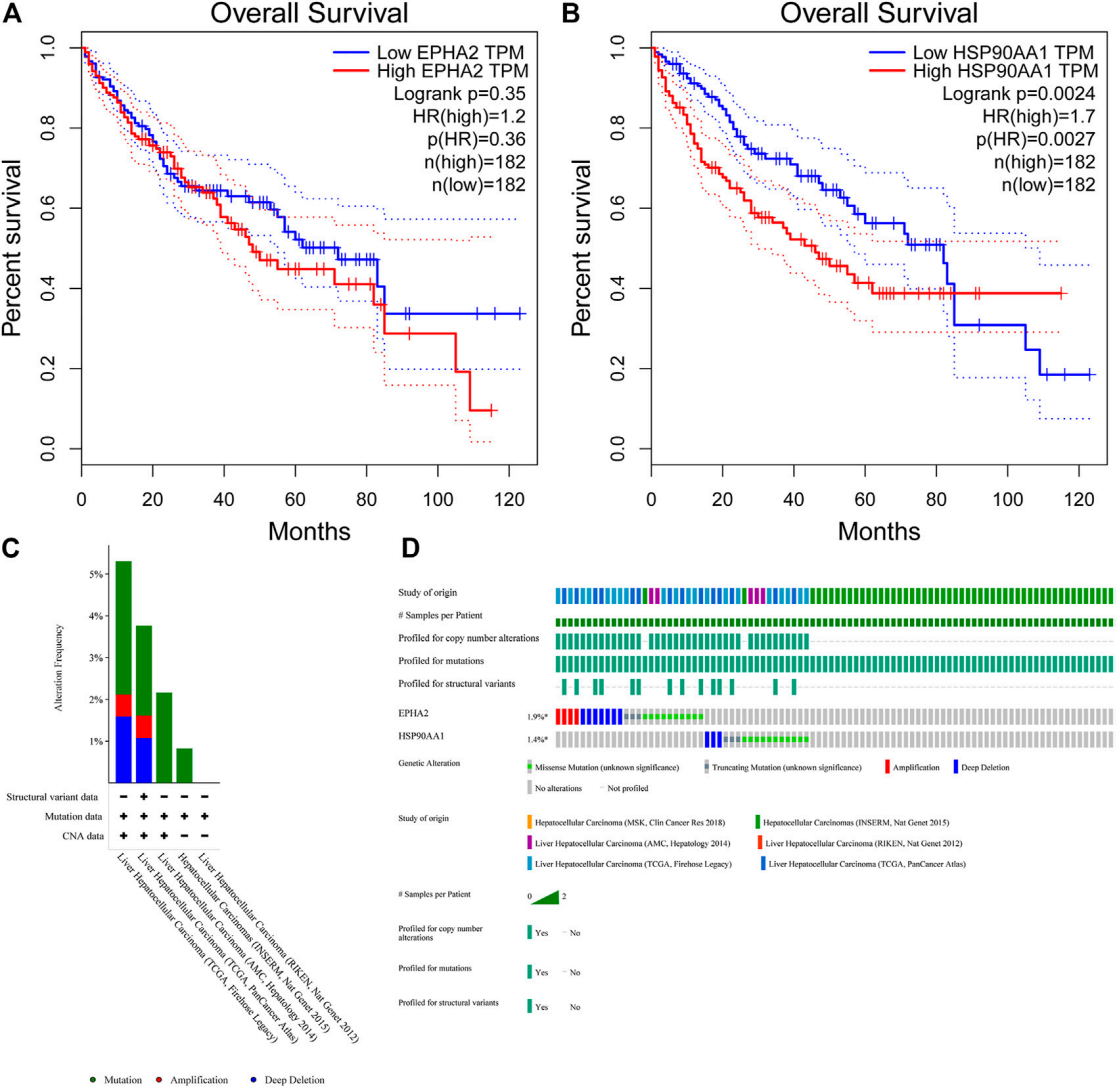
**(A, B)** Survival analysis of high and low expression populations of EPHA2 and HSP90AA1. (In the figure, the red curve refers to the population with high expression in the TCGA database, and the blue curve refers to the population with low expression in the TCGA database. The P value represents the statistical difference in the survival curve between the two groups). **(C, D)** Genetic mutation analysis of EPHA2 and HSP90AA1 in various TCGA datasets. **(C)** The percentage of total mutations of EPHA2 and HSP90AA1. **(D)** The mutations of EPHA2 and HSP90AA1 in each sample.

The TIMER database showed that EPHA2 and HSP90AA1 were differentially expressed in multiple tumors, and EPHA2 was expressed at low levels in HCC. The mRNA expression levels of EPHA2 and HSP90AA1 were strongly correlated with the DNA methylation level (Figure 6), suggesting that the regulatory effect of GJF on EPHA2 and HSP90AA1 might be caused by the regulation of their methylation status by methyltransferase and then the regulation of their expression. However, HSP90AA1 was hypermethylated and highly expressed in HCC, and there was a negative correlation between the two, which may require further experimental confirmation. Our study showed that low expression of EPHA2 may have a better prognosis, but the difference was not statistically significant (Figure 7A, *p* = 0.35). In multiple TCGA datasets, the mutation rate of EPHA2 was 1.9% and that of HSP90AA1 was 1.4% (Figures 7C, D), suggesting that these two molecules have a certain application value, but further basic experiments may be needed to confirm this.

### 3.7 Effects on cell proliferation and migration

**FIGURE 8:**
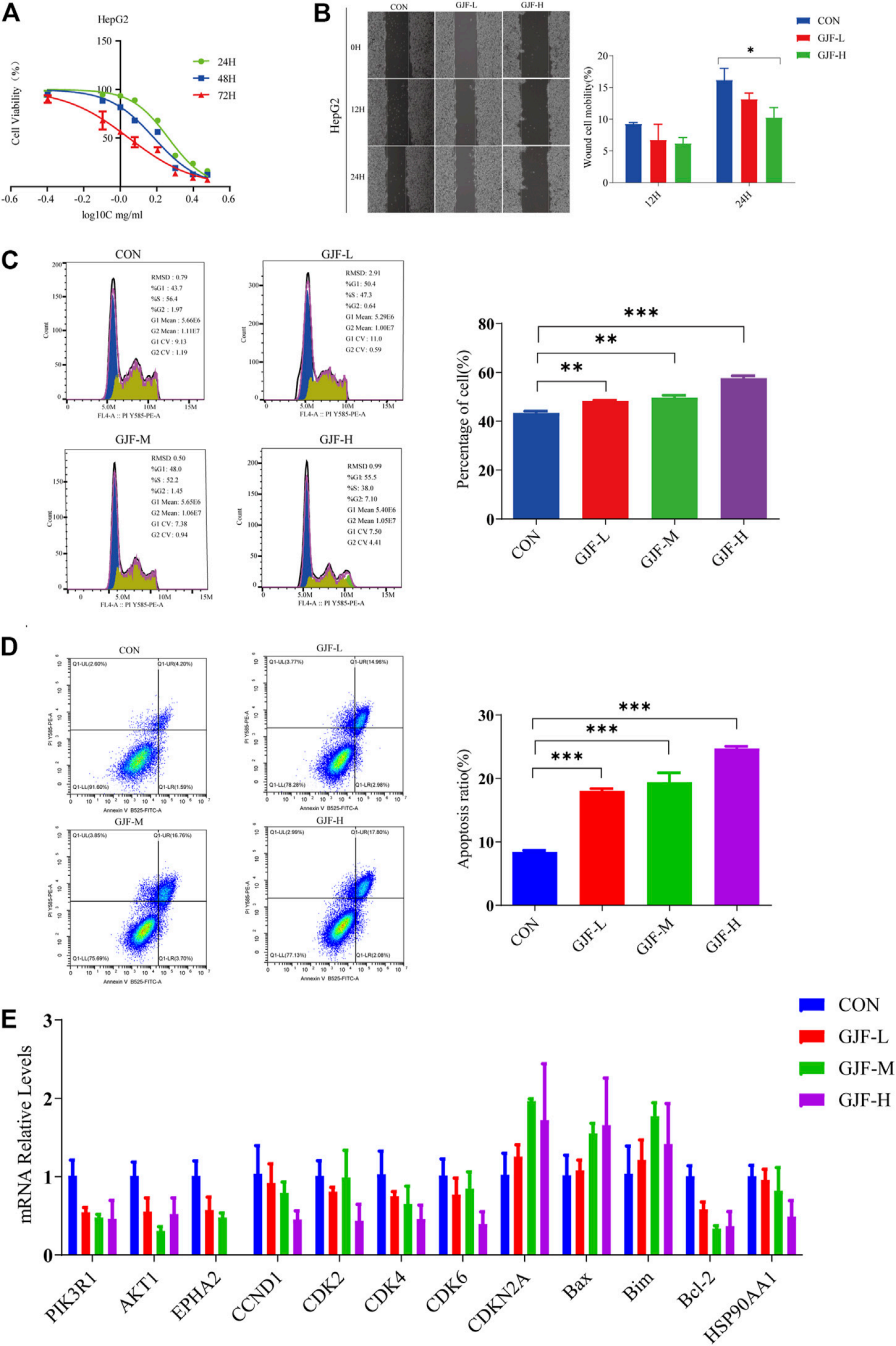
GJF inhibited HepG2 cells growth *in vitro*. **(A)** The effect of GJF on the survival rate of HepG2 cells. **(B)** Effect of GJF on the cell mobility of HepG2 cells. **(C)** GJF induces hepatocellular arrest of HepG2 cells cycles. **(D)** GJF induces apoptosis in HepG2 cells. **(E)** Changes in the mRNA expression levels of related molecules in the PI3K/Akt pathway in HepG2 cell lines. Gene expression was evaluated by RT-qPCR, using the 2 −∆∆CT method. (CON, negative control treatment; GJF-L, treated with low concentrations (0.5 mg/ml) of GJF; GJF-M, treated with medium concentrations (1.0 mg/ml) of GJF; GJF-H, treated with medium concentrations (1.5 mg/ml) of GJF. Data are presented as the mean ± SD, and analysis of variance was used to determine the significance. *p < 0.05; **p < 0.01; ***p < 0.001 compared with the control group).

The MTT experiment results showed that a certain concentration of GJF freeze-dried powder solution inhibited the proliferation of HepG2 cell strains. With increasing drug concentration and time, the inhibition gradually increased in a time- and concentration-dependent manner (Figure 8A). The IC_50_ values of 24H, 48H, and 72H in the HepG2 cell line were 1.91 mg/ml, 1.56 mg/ml, and 1.12 mg/ml, respectively. The GJF freeze-dried powder solution was divided into a low concentration group (0.5 mg/ml), medium concentration group (1.0 mg/ml), and high concentration group (3.0 mg/ml).

The wound healing assay showed that compared with the control group, the cell mobility of the high concentration group and the low concentration group showed a downward trend in the HepG2 cell line. After 24 h of treatment, the inhibitory effect of the high concentration group was significantly stronger than that of the control group (Figure 8B).

### 3.8 Effects of the cell cycle and apoptosis

The cell cycle can be divided into the GOG1 phase, G phase, and G2/M phase, and the ratio of the GOG1 phase is usually used to represent its inhibitory effect on proliferation. As shown in the figure below, GJF blocked HepG2 cell lines in the GOG1 phase in a concentration-dependent manner (Figure 8C). Compared with the control group, the difference between the low-dose, medium-dose, and high-dose groups and the control group was statistically significant.

As shown in Figure 8D, GJF blocked HepG2 cell lines in the G0/G1 phase in a concentration-dependent manner. In the HepG2 cell line, the apoptosis rate of the medium-dose group was higher than that of the high-dose group. Compared with the control group, the differences between the low-dose group, medium-dose group, and high-dose group and the control group were statistically significant.

### 3.9 Validation of related indicators in the PI3K/Akt signaling pathway

We verified the relevant molecules in the PI3K/Akt signaling pathway in the HepG2 cell line (Supplementary Table S3). According to the RT‒qPCR results (Figure 8E), we observed that GJF reduced the expression levels of PIK3R1 and Akt1 in the HepG2 cell line, and the difference was statistically significant. These results suggest that GJF may inhibit the expression level of the PI3K/Akt signaling axis in HCC. On the other hand, GJF decreased the levels of cycling-related proteins (CCND1, CDK2, CDK4, and CDK6) in HepG2 cell lines and increased the expression of the tumor suppressor gene CDKN2A. For apoptosis-related proteins, GJF increased the expression levels of Bax and Bim while decreased the expression level of Bel-2. In the HepG2 cell line, GJF decreased the expression levels of EPHA2.

## 4 Discussion

HCC, the most prevalent type of PLC, is a common malignant tumor threatening human health and life (Siegel et al., 2020). Clinically, the combination of traditional Chinese and Western medicine is very important in the treatment of HCC. Preliminary clinical and animal studies have explored the effects of GJF on anti-inflammatory, antitumor, liver protection, inhibition of tumor cell proliferation, and induction of tumor cell apoptosis. Therefore, this study further explored the clinical application value and possible mechanism of action of GJF along with previous research ideas.

To further explore the mechanism of GJF intervention in the HCC process, we first screened the potential active compounds of GJF acting on HCC and clarified the molecular mechanism of GJF intervention in HCC through the PI3K/Akt signaling axis using network pharmacology. A total of 63 key targets of GJF acting on HCC were obtained, and the results showed that the top five BPs involved in these genes were epithelial cell proliferation, regulation of epithelial cell proliferation, regulation of cell cycle phase transition, gland development, and embryonic organ development, respectively; In terms of CC, the targets were mainly involved in cell-substrate junction, cell-substrate adherens junction, focal adhesion, adherens junction, and extracellular matrix composition. In the category of MF, they were mainly associated with cell adhesion molecule binding, proximal promoter sequence-specific DNA binding, chromatin binding, RNA polymerase II proximal promoter sequence-specific DNA binding and laminin-binding are related. In addition, the top five pathways were the cell cycle, cellular senescence, p53 signaling pathway, PI3K/Akt signaling pathway, and progesterone-mediated oocyte maturation. Studies have shown that telomere shortening and cell cycle checkpoint inactivation are characteristic of the development of human liver cancer (Plentz et al., 2007). Cellular senescence is the process leading to end-of-life growth arrest with characteristic morphological features, mediated by telomere dependent, oncogene-induced, and ROS-induced pathways, but persistent DNA damage is the most common cause. Aging arrest is mediated by p16-and p21-dependent pathways that both lead to retinoblastoma protein activation. p53 acts as a relay between DNA damage detection and p21 activation, and aging arrest and cellular immortality are likely to significantly promote the development of HCC. Aging in oncogene-induced precancerous lesions and reversible immortality of cancer cells, including HCCs, offer new potential for tumor prevention and treatment (Ozturk et al., 2009). Yan Wang et al. showed that circulating neutrophils can predict low HCC survival and promote HCC progression through the p53 and STAT3 signaling pathways (Wang et al., 2020). Several studies have shown that progestin-mediated oocyte maturation is strongly associated with the progression of HCC, but further experimental confirmation is needed (B. Jin et al., 2015; Ma et al., 2020). Xiao-Lu Ma et al. showed that CD73 could promote the progression and metastasis of HCC by inducing RAP1-mediated P110β membrane localization to activate PI3K/Akt signaling, which was associated with poor prognosis of HCC(X. L. Ma et al., 2019). Similarly, the results showed that GJF may regulate the mRNA expression levels of relevant molecules in the PI3K/Akt signaling pathway and play a role in regulating the mRNA expression levels of proteins related to proliferation and apoptosis downstream of the pathway.

HSP90AA1 plays an important role in the proliferation, differentiation, survival, and angiogenesis of tumor cells (Jego et al., 2013). HSP90AA1 is highly expressed in a variety of malignancies, including breast, endometrial, ovarian, colon, lung, and prostate cancers (Kang et al., 2010; Y; Li et al., 2011; Soroka et al., 2012), which is consistent with our conclusion based on the TCGA database. Qiuran Xu et al. showed that Hsp90 can regulate the abundance of PKM2 through phosphorylation of Thr-328 to promote cell glycolysis and proliferation and inhibit apoptosis of HCC cells (Q. Xu et al., 2017). Xiao Xiang et al. showed that the VEGF/VEGFR2 pathway may be related to the recurrence of HCC in patients with high expression of HSP90AA1 (Xiang et al., 2018). EPHA2 overexpression is associated with tumor progression, metastasis and prognosis of HCC. Pu Yang et al. showed that EPHA2 is involved in key mediators of angiogenesis and invasion (Yang et al., 2009). Qiao Jin et al. showed that EPHA2 can negatively regulate the radiosensitivity of MHCC97H cells (Jin et al., 2015), which is consistent with our findings. We verified the application value of EPHA2 by analyzing data from the TCGA database, and the results showed that EPHA2 was differentially expressed in a variety of tumors, and its high expression was associated with poor prognosis in HCC, but the difference between the high and low expression of EPHA2 was not statistically significant. The RT‒qPCR results showed that GJF decreased the mRNA expression levels of EPHA2 in HepG2 cells, and the expression levels decreased gradually with increasing hepatocellular formula concentration. The mRNA expression level of HSP90AA1 in HepG2 cells decreased gradually with increasing GJF concentration but only in the high-dose group (*p* < 0.05). Our hypothesis was further verified by RT‒qPCR results. GJF may inhibit the mRNA expression levels of related molecules in the PI3K/Akt signaling pathway and regulate the mRNA expression levels of proliferation- and apoptosis-related proteins downstream of the pathway. Combined with molecular docking technology, EPHA2 may be the key target of GJF in HCC through the PI3K/Akt signaling pathway.

Molecular docking results showed that PF and PA might be the key compounds in hepatocellular formation and have anticancer activity. PF is an important compound that protects the liver, reduces cholestasis, alleviates liver fibrosis, and prevents HCC(X. Ma et al., 2020). Yang Zhou et al. showed that PF may block the expression of the Wnt/β-catenin pathway by downregulating the expression of 5-HT1D, thus inhibiting the occurrence of HCC(Zhou et al., 2020). In addition, PF is an effective antimetastatic and anti-invasive agent for inhibiting HCC invasion and metastasis (J. T. Lu et al., 2014). PA is a triterpenoid compound with anticancer activity that has anti-inflammatory, anticancer, anti-aging, and insulin-like properties and other pharmacological effects (Miao et al., 2019). It induces apoptosis in human prostate cancer (Gapter et al., 2005), lung cancer (C. Lu et al., 2018), and bladder cancer (Jeong et al., 2015) cells. In conclusion, our results suggest that PA and PF may be the key compounds of hepatocellular development that exert anticancer activity.

However, our study had certain limitations. Because TCM has the characteristics of multitarget and multipathway effects, the pathway we verified is likely to be only one way for GJF to exert its effects. In this study, we made predictions based on data analysis and a small number of *in vitro* experiments. GJF is a well-known prescription with beneficial properties. Therefore, further *in vivo* and *in vitro* experimental verification is still necessary.

## 5 Conclusion

In conclusion, this study revealed a potential pharmacological mechanism by which GJF can treat HCC at the systemic level, possibly involving the synergistic effects of multiple mechanisms, such as cell proliferation, apoptosis, cell migration, immune regulation, and inflammatory induction. Our results suggested that GJF may induce G0/G1 phase cycle arrest by inhibiting the PI3K/Akt signaling pathway and promoting the apoptosis of liver cancer cell lines. EPHA2 may be an important target of GJF in HCC, and pachymic acid may be an important active compound of GJF that exerts anticancer activity. This study can provide a scientific basis for the subsequent clinical application of GJF and the in-depth study of its mechanism. Given that the study is primarily based on data analysis, further biological experiments are crucial to verify the results.

## Data Availability

The datasets presented in this study can be found in online repositories. The names of the repository/repositories and accession number(s) can be found in the article/Supplementary Material.

## Supplementary material

Supplementary Table 1 Top 5 compounds in GJF (ranked by OB %).

Supplementary Table 2 Top 10 KEGG Pathways in which GJF acted on the key targets of HCC (ranked by number of genes enriched).

Supplementary Table 3 mRNA expression level of each molecule in HepG2 cell line.
