# Supplementary material for: Molecular mechanism of Ganji Fang in the treatment of hepatocellular carcinoma based on network pharmacology, molecular docking and experimental verification technology
Source: Front Pharmacol. 2023 Jan 19;14:1016967. doi: 10.3389/fphar.2023.1016967 (PMC9892186; doi:10.3389/fphar.2023.1016967)
Supplement: Supplementary file 1 [file Table1.DOCX]

**Supplementary Table 1 Top 5 compounds in GJF (ranked by OB %)**

| **Herb** | **Mol ID** | **Molecule Name** | **Molecules Structure** | **OB (%)** | **DL** |
| --- | --- | --- | --- | --- | --- |
| Radix Bupleuri | MOL004644 | Sainfuran | 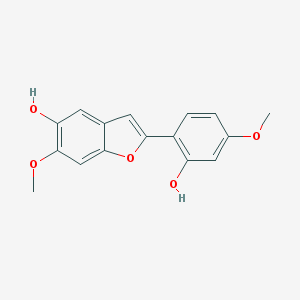 | 79.91 | 0.23 |
| Radix Bupleuri | MOL013187 | Cubebin | 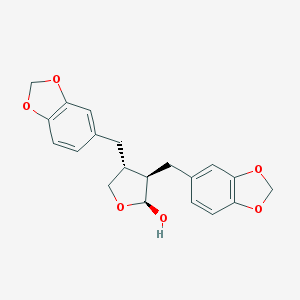 | 57.13 | 0.64 |
| Radix Bupleuri | MOL000354 | isorhamnetin | 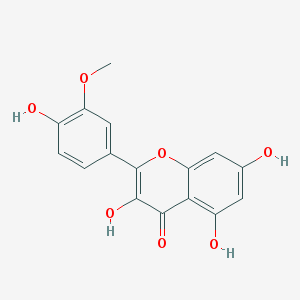 | 49.6 | 0.31 |
| Radix Bupleuri | MOL004609 | Areapillin | 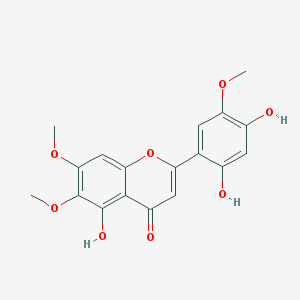 | 48.96 | 0.41 |
| Radix Bupleuri | MOL004628 | Octalupine | 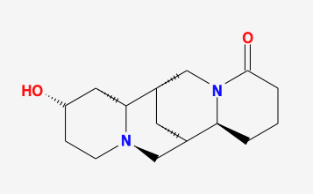 | 47.82 | 0.28 |
| Scutellariae Radix | MOL002934 | NEOBAICALEIN | 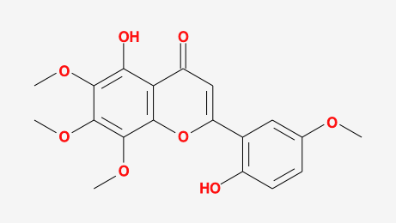 | 104.34 | 0.44 |
| Scutellariae Radix | MOL002932 | Panicolin | 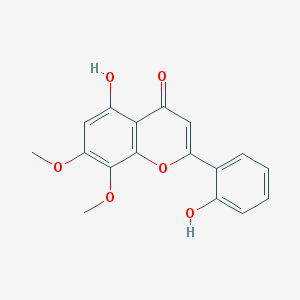 | 76.26 | 0.29 |
| Scutellariae Radix | MOL012246 | 5,7,4'-trihydroxy-8-methoxyflavanone | 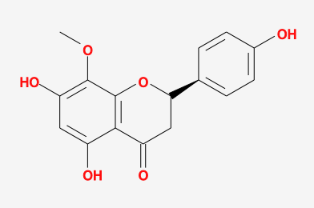 | 74.24 | 0.26 |
| Scutellariae Radix | MOL002927 | Skullcapflavone II | 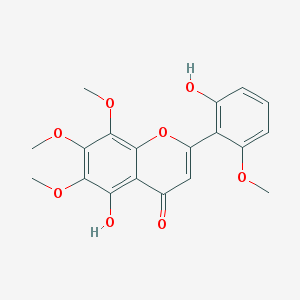 | 69.51 | 0.44 |
| Scutellariae Radix | MOL002911 | 2,6,2',4'-tetrahydroxy-6'-methoxychaleone | 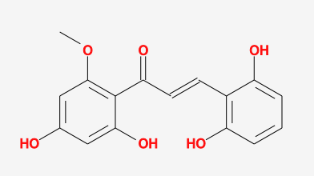 | 69.04 | 0.22 |
| Codonopsitis Radix | MOL002140 | Perlolyrine | 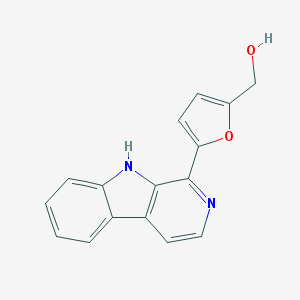 | 65.95 | 0.27 |
| Codonopsitis Radix | MOL005321 | Frutinone A | 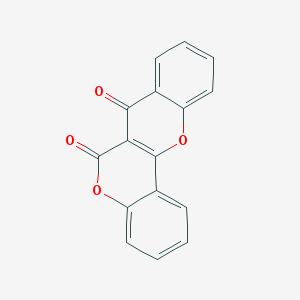 | 65.9 | 0.34 |
| Codonopsitis Radix | MOL008400 | glycitein | 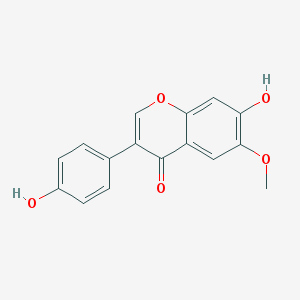 | 50.48 | 0.24 |
| Codonopsitis Radix | MOL008397 | Daturilin | 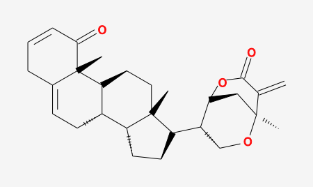 | 50.37 | 0.77 |
| Codonopsitis Radix | MOL008407 | (8S,9S,10R,13R,14S,17R)-17-[(E,2R,5S)-5-ethyl-6-methylhept-3-en-2-yl]-10,13-dimethyl-1,2,4,7,8,9,11,12,14,15,16,1-dodecahydrocyclopenta[a]phenanthren-3-one | 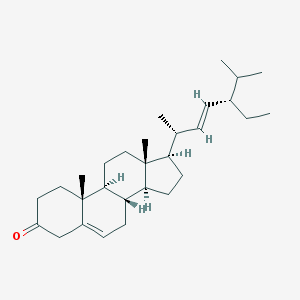 | 45.4 | 0.76 |
| Paeoniae Radix Alba | MOL001918 | paeoniflorgenone | 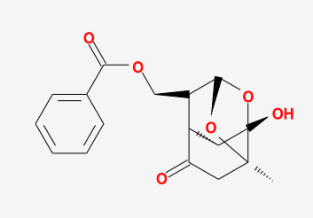 | 87.59 | 0.37 |
| Paeoniae Radix Alba | MOL001925 | paeoniflorin_qt | 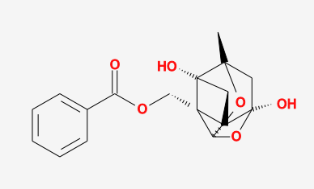 | 68.18 | 0.4 |
| Paeoniae Radix Alba | MOL001928 | albiflorin_qt | 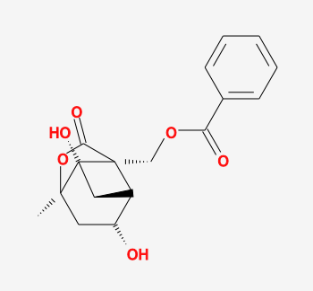 | 66.64 | 0.33 |
| Paeoniae Radix Alba | MOL001910 | 11alpha,12alpha-epoxy-3beta-23-dihydroxy-30-norolean -20-en-28,12beta-olide | 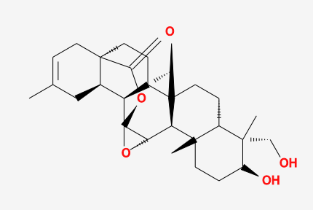 | 64.77 | 0.38 |
| Paeoniae Radix Alba | MOL000211 | Mairin | 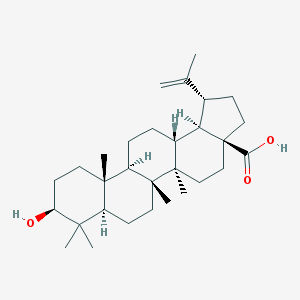 | 55.38 | 0.78 |
| Poria cocos | MOL000300 | dehydroeburicoic acid | 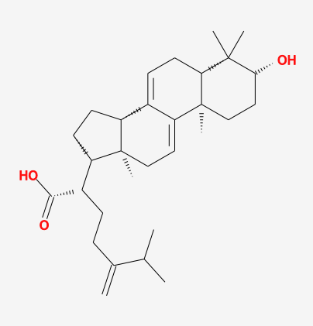 | 44.17 | 0.83 |
| Poria cocos | MOL000282 | ergosta-7,22E-dien-3beta-ol | 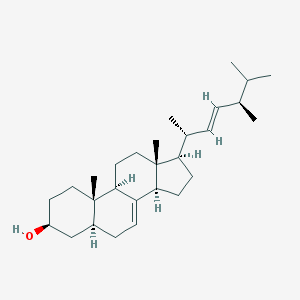 | 43.51 | 0.72 |
| Poria cocos | MOL000283 | Ergosterol peroxide | 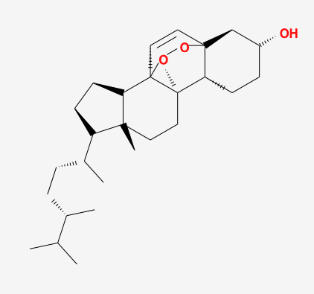 | 40.36 | 0.81 |
| Poria cocos | MOL000275 | trametenolic acid | 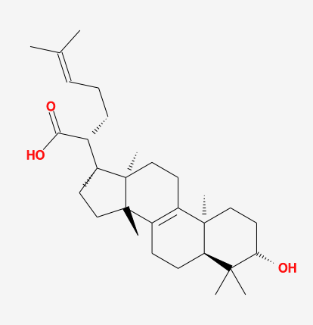 | 38.71 | 0.8 |
| Poria cocos | MOL000287 | 3beta-Hydroxy-24-methylene-8-lanostene-21-oic acid | 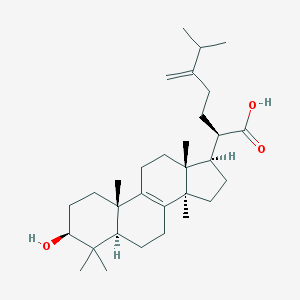 | 38.7 | 0.81 |
| Atractylodes Macrocephala Koidz | MOL000022 | 14-acetyl-12-senecioyl-2E,8Z,10E-atractylentriol | 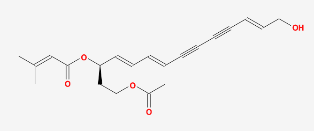 | 63.37 | 0.3 |
| Atractylodes Macrocephala Koidz | MOL000020 | 12-senecioyl-2E,8E,10E-atractylentriol | 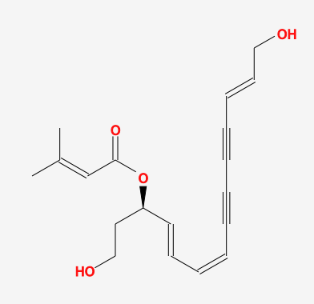 | 62.4 | 0.22 |
| Atractylodes Macrocephala Koidz | MOL000021 | 14-acetyl-12-senecioyl-2E,8E,10E-atractylentriol | 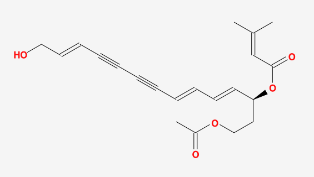 | 60.31 | 0.31 |
| Atractylodes Macrocephala Koidz | MOL000049 | 3β-acetoxyatractylone | 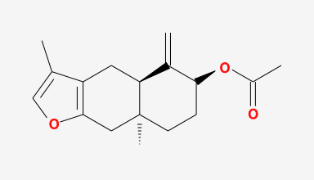 | 54.07 | 0.22 |
| Atractylodes Macrocephala Koidz | MOL000028 | α-Amyrin | 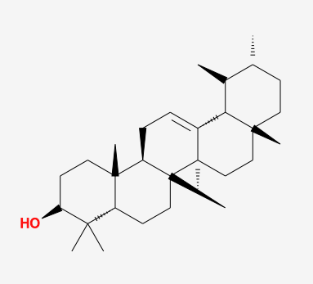 | 39.51 | 0.76 |
| Akebiae Frucyus | MOL010929 | glyceryl linolenate | 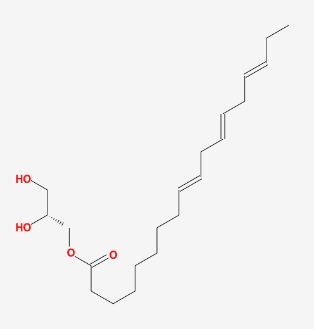 | 38.14 | 0.31 |
| Akebiae Frucyus | MOL000358 | beta-sitosterol | 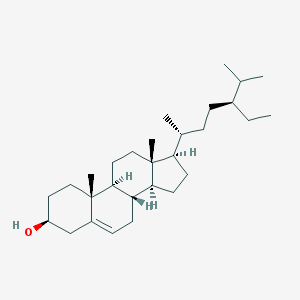 | 36.91 | 0.75 |
| Akebiae Frucyus | MOL000359 | sitosterol | 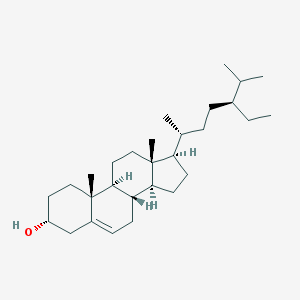 | 36.91 | 0.75 |
| Akebiae Frucyus | MOL008121 | 2-Monoolein | 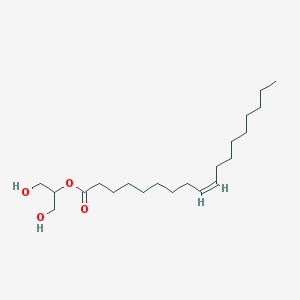 | 34.23 | 0.29 |
| Akebiae Frucyus | MOL002882 | [(2R)-2,3-dihydroxypropyl] (Z)-octadec-9-enoate | 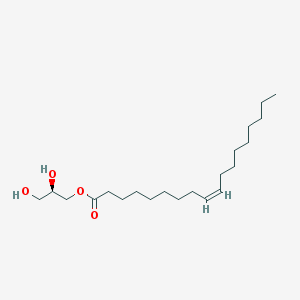 | 34.13 | 0.3 |
| Curcumae Rhizoma | MOL000940 | bisdemethoxycurcumin | 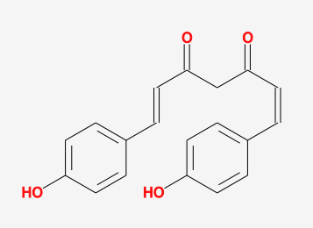 | 77.38 | 0.26 |
| Curcumae Rhizoma | MOL000906 | wenjine | 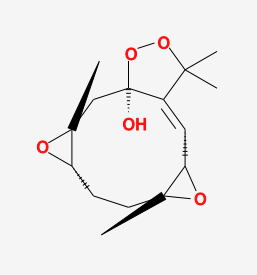 | 47.93 | 0.27 |
| Curcumae Rhizoma | MOL000296 | hederagenin | 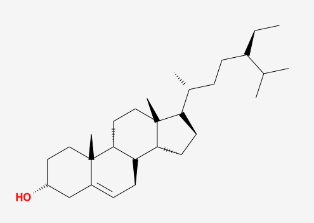 | 36.91 | 0.75 |
| Curcumae Rhizoma | MOL000295 | alexandrin | 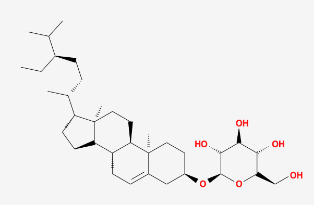 | 20.63 | 0.63 |
| Curcumae Rhizoma | MOL000893 | difurocumenone | 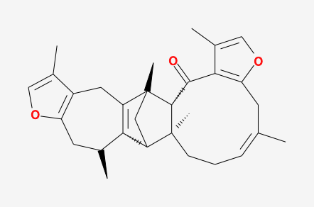 | 8.86 | 0.61 |
| Scutellariae Barbatae Herba | MOL012246 | 5,7,4'-trihydroxy-8-methoxyflavanone | 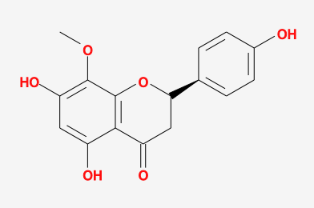 | 74.24 | 0.26 |
| Scutellariae Barbatae Herba | MOL005190 | eriodictyol | 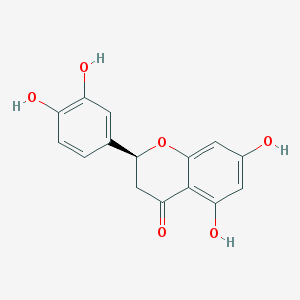 | 71.79 | 0.24 |
| Scutellariae Barbatae Herba | MOL012248 | 5-hydroxy-7,8-dimethoxy-2-(4-methoxyphenyl) chromone | 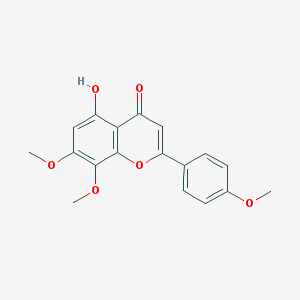 | 65.82 | 0.33 |
| Scutellariae Barbatae Herba | MOL002915 | Salvigenin | 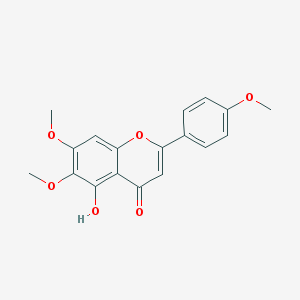 | 49.07 | 0.33 |
| Scutellariae Barbatae Herba | MOL000351 | Rhamnazin | 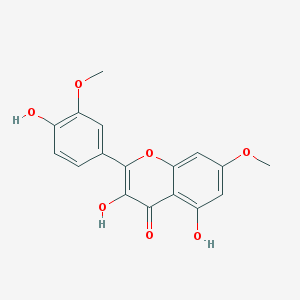 | 47.14 | 0.37 |
| Ecliptae Herba | MOL003402 | demethylwedelolactone | 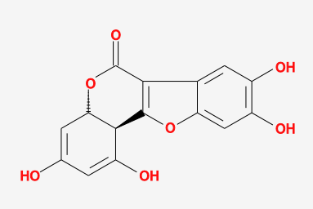 | 72.13 | 0.43 |
| Ecliptae Herba | MOL002975 | butin | 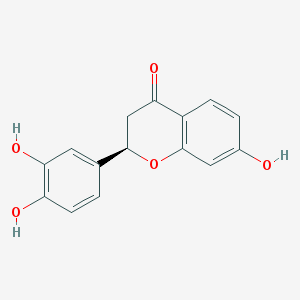 | 69.94 | 0.21 |
| Ecliptae Herba | MOL003389 | 3'-O-Methylorobol | 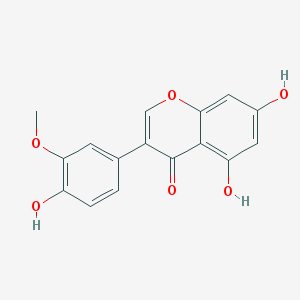 | 57.41 | 0.27 |
| Ecliptae Herba | MOL003404 | wedelolactone | 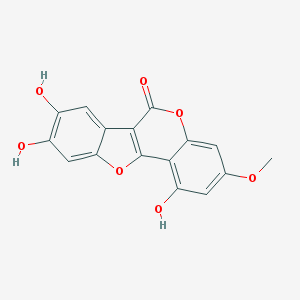 | 49.6 | 0.48 |
| Ecliptae Herba | MOL000098 | quercetin | 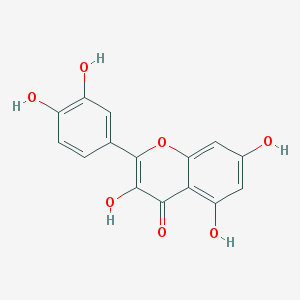 | 46.43 | 0.28 |
| Fructus Ligustri Lucidi | MOL005212 | Olitoriside_qt | 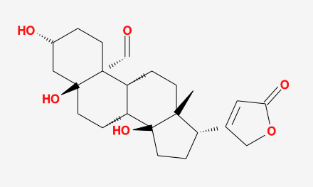 | 103.23 | 0.78 |
| Fructus Ligustri Lucidi | MOL005195 | syringaresinol diglucoside_qt | 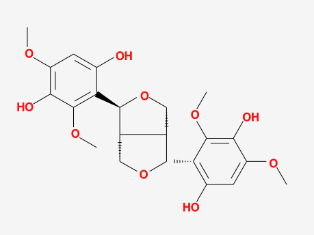 | 83.12 | 0.8 |
| Fructus Ligustri Lucidi | MOL005190 | eriodictyol | 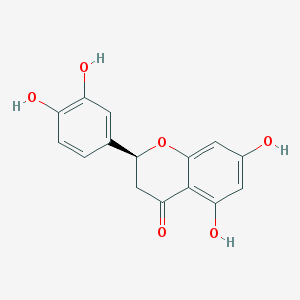 | 71.79 | 0.24 |
| Fructus Ligustri Lucidi | MOL005211 | Olitoriside | 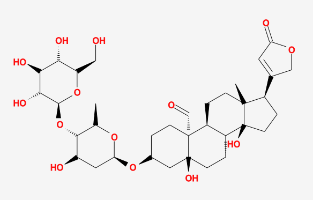 | 65.45 | 0.23 |
| Fructus Ligustri Lucidi | MOL004576 | taxifolin | 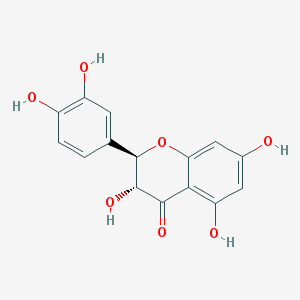 | 57.84 | 0.27 |
| Herba Sarcandrae | MOL007132 | (2R)-3-(3,4-dihydroxyphenyl)-2-[(Z)-3-(3,4-dihydroxyphenyl) acryloyl]oxy-propionic acid | 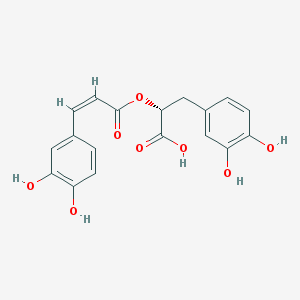 | 109.38 | 0.35 |
| Herba Sarcandrae | MOL007747 | chloranoside a_qt | 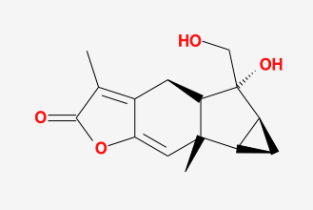 | 84.11 | 0.23 |
| Herba Sarcandrae | MOL007742 | Istanbulin-A | 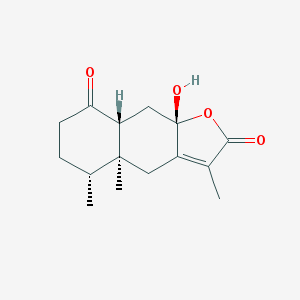 | 80.1 | 0.2 |
| Herba Sarcandrae | MOL000098 | quercetin | 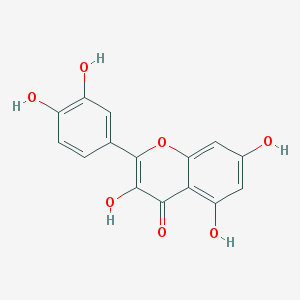 | 46.43 | 0.28 |
| Herba Sarcandrae | MOL004373 | Anhydroicaritin | 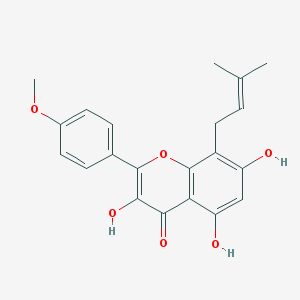 | 45.41 | 0.44 |
