# Supplementary material for: Molecular mechanism of Ganji Fang in the treatment of hepatocellular carcinoma based on network pharmacology, molecular docking and experimental verification technology
Source: Front Pharmacol. 2023 Jan 19;14:1016967. doi: 10.3389/fphar.2023.1016967 (PMC9892186; doi:10.3389/fphar.2023.1016967)
Supplement: Supplementary file 2 [file Table2.DOCX]

**Supplementary Table 2 Top 10 KEGG Pathways in which GJF acted on the key targets of HCC (ranked by number of genes enriched)**

| ID | Description | p-value | p.adjust | q-value | geneID | Count |
| --- | --- | --- | --- | --- | --- | --- |
| hsa04110 | Cell cycle | 9.76E-10 | 1.23E-07 | 1.05E-07 | CCNE1/CCND2/CCNA2/CDK1/TTK/CDK4/CDC25A/CCNB2/PLK1/CDK6/CDK2 | 11 |
| hsa04218 | Cellular senescence | 1.79E-07 | 8.34E-06 | 7.09E-06 | CCNE1/CCND2/CCNA2/IGFBP3/CDK1/CDK4/CDC25A/CCNB2/CDK6/CDK2 | 10 |
| hsa04115 | p53 signaling pathway | 1.76E-09 | 1.23E-07 | 1.05E-07 | CCNE1/CCND2/IGFBP3/CDK1/CDK4/CASP8/CCNB2/CDK6/CDK2 | 9 |
| **hsa04151** | **PI3K-Akt signaling pathway** | **0.000938604** | **0.021900758** | **0.018607411** | **CCNE1/PKN1/CCND2/CDK4/EPHA2/FGFR3/CDK6/CDK2/HSP90AA1** | **9** |
| hsa04914 | Progesterone-mediated oocyte maturation | 4.86E-07 | 1.70E-05 | 1.45E-05 | CCNA2/CDK1/AURKA/CDC25A/CCNB2/PLK1/CDK2/HSP90AA1 | 8 |
| hsa04114 | Oocyte meiosis | 0.00031378 | 0.008785829 | 0.007464652 | CCNE1/CDK1/AURKA/CCNB2/PLK1/CDK2 | 6 |
| hsa04066 | HIF-1 signaling pathway | 0.001133913 | 0.022678253 | 0.01926799 | HK1/NOS2/PGK1/SLC2A1/PLCG1 | 5 |
| hsa04915 | Estrogen signaling pathway | 0.003207315 | 0.056128016 | 0.047687713 | PRKCD/HSPA1A/SRC/MMP9/HSP90AA1 | 5 |
| hsa04621 | NOD-like receptor signaling pathway | 0.010007347 | 0.127078588 | 0.107969025 | PRKCD/PKN1/CASP8/TYK2/HSP90AA1 | 5 |
| hsa04919 | Thyroid hormone signaling pathway | 0.01089245 | 0.127078588 | 0.107969025 | SRC/SLC2A1/ATP1A1/PLCG1 | 4 |
