# Supplementary material for: Molecular mechanism of Ganji Fang in the treatment of hepatocellular carcinoma based on network pharmacology, molecular docking and experimental verification technology
Source: Front Pharmacol. 2023 Jan 19;14:1016967. doi: 10.3389/fphar.2023.1016967 (PMC9892186; doi:10.3389/fphar.2023.1016967)
Supplement: Supplementary file 3 [file Table3.DOCX]

**Supplementary Table 3** mRNA expression level of each molecule in HepG2 cell line

| Gene name | Grouping | Repeat number | Average | Standard deviation | | P values |
| --- | --- | --- | --- | --- | --- | --- |
| PIK3R1 | C | 3 | 1.0126 | 0.19906 |  | |
|  | L | 3 | 0.5464 | 0.05991 | 0.007 | |
|  | M | 3 | 0.4777 | 0.03987 | 0.003 | |
|  | H | 3 | 0.4623 | 0.23491 | 0.003 | |
| AKT1 | C | 3 | 1.01 | 0.17692 |  | |
|  | L | 3 | 0.5533 | 0.17243 | 0.009 | |
|  | M | 3 | 0.31 | 0.05196 | 0.001 | |
|  | H | 3 | 0.5233 | 0.20502 | 0.006 | |
| EPHA2 | C | 3 | 1.0133 | 0.19035 |  | |
|  | L | 3 | 0.5733 | 0.16442 | 0.003 | |
|  | M | 3 | 0.48 | 0.06083 | 0.001 | |
|  | H | 3 | 1.01098809 | .10461187 | .000 | |
| CCND1 | C | 3 | 1.038 | 0.3584 |  | |
|  | L | 3 | 0.9209 | 0.24384 | 0.557 | |
|  | M | 3 | 0.7947 | 0.13723 | 0.238 | |
|  | H | 3 | 0.4539 | 0.1098 | 0.016 | |
| CDK2 | C | 3 | 1.0133 | 0.19009 |  | |
|  | L | 3 | 0.8067 | 0.05508 | 0.286 | |
|  | M | 3 | 0.9933 | 0.33946 | 0.915 | |
|  | H | 3 | 0.4367 | 0.20502 | 0.013 | |
| CDK4 | C | 3 | 1.0333 | 0.32005 |  | |
|  | L | 3 | 0.9433 | 0.19858 | 0.607 | |
|  | M | 3 | 0.7467 | 0.07371 | 0.127 | |
|  | H | 3 | 0.5 | 0.15 | 0.013 | |
| CDK6 | C | 3 | 1.0159 | 0.2114 |  | |
|  | L | 3 | 0.7702 | 0.21015 | 0.169 | |
|  | M | 3 | 0.8484 | 0.21333 | 0.333 | |
|  | H | 3 | 0.3956 | 0.15623 | 0.005 | |
| CDKN2A | C | 3 | 1.0229 | 0.27512 |  | |
|  | L | 3 | 1.2571 | 0.14988 | 0.486 | |
|  | M | 3 | 1.9668 | 0.02742 | 0.019 | |
|  | H | 3 | 1.7226 | 0.71955 | 0.061 | |
| Bax | C | 3 | 1.0196 | 0.25479 |  | |
|  | L | 3 | 1.0801 | 0.13032 | 0.088 | |
|  | M | 3 | 1.5549 | 0.12521 | 0.049 | |
|  | H | 3 | 1.6599 | 0.59791 | 0.832 | |
| Bim | C | 3 | 1.0376 | 0.35461 |  | |
|  | L | 3 | 1.2132 | 0.25663 | 0.554 | |
|  | M | 3 | 1.7734 | 0.17058 | 0.032 | |
|  | H | 3 | 1.4193 | 0.51506 | 0.217 | |
| Bcl-2 | C | 3 | 1.0056 | 0.1333 |  | |
|  | L | 3 | 0.5831 | 0.09378 | 0.003 | |
|  | M | 3 | 0.3358 | 0.03845 | 0 | |
|  | H | 3 | 0.3691 | 0.18533 | 0 | |
| HSP90AA1 | C |  | 1.0059456 | 0.13718412 |  | |
|  | L | 3 | 0.9596876 | 0.13514722 | 0.788 | |
|  | M | 3 | 0.8206474 | 0.29622073 | 0.297 | |
|  | H | 3 | 0.4911238 | 0.20199447 | 0.015 | |

Note: C, treated with negative control; GJF-L, treated with low concentrations (0.5 mg/ml) of GJF; M, treated with medium concentrations (1.0 mg/ml) of GJF; H, treated with medium concentrations (1.5 mg/ml) of GJF. Data are presented as the mean ± SD, analysis of variance were used to determine the significance. *p < 0.05; **p < 0.01; ***p < 0.001 compared with the control group.
